# “In our culture, if you quarantine someone, you stigmatize them”: Qualitative insights on barriers to observing COVID-19 prevention behaviors in Côte d’Ivoire

**DOI:** 10.1371/journal.pgph.0000489

**Published:** 2022-08-24

**Authors:** Natalie Jean Tibbels, Abdul Dosso, Kouamé Walter Kra, Konan Dorgeles Gbeke, Gervais Coffi, Alex Romeo Ngoran, Jean Louis Niamke, Marjorie Nana, William Benié, Zoé Mistrale Hendrickson, Danielle Amani Naugle

**Affiliations:** 1 Center for Communication Programs, Health, Behavior & Society Department, Johns Hopkins Bloomberg School of Public Health, Baltimore, Maryland, United States of America; 2 Center for Communication Programs-Côte d’Ivoire, Johns Hopkins Bloomberg School of Public Health, Abidjan, Côte d’Ivoire; 3 Department of Sociology, Université Alassane Ouattara, Bouaké, Côte d’Ivoire; 4 Department of Sociology, Université de Cocody, Abidjan, Côte d’Ivoire; University of Vienna, AUSTRIA

## Abstract

While vaccines are now authorized for use against the SARS-CoV2 virus, they remain inaccessible for much of the world and widespread hesitancy persists. Ending the COVID-19 pandemic depends on continued prevention behaviors such as mask wearing, distancing, hand hygiene, and limiting large gatherings. Research in low- and middle-income countries has focused on the prevalence of adherence and demographic determinants, but there is a need for a nuanced understanding of why people do or do not practice a given prevention behavior. The Breakthrough ACTION project led by Johns Hopkins Center for Communication Programs conducted a qualitative study in November 2020 in Côte d’Ivoire to explore people’s experience with and perceptions of the COVID-19 pandemic. We conducted 24 focus group discussions and 29 in-depth interviews with members of the general population and health providers. This analysis explores barriers and facilitators to seven recommended prevention behaviors with a particular focus on response efficacy, self-efficacy, and social norms. We found these constructs to be salient for participants who generally felt that the behaviors were useful for preventing COVID-19 but were difficult to practice for a variety of reasons. The perception that COVID-19 prevention behaviors were anti-social emerged as a key theme. Behavior change interventions must reframe the recommended behaviors as pro-social, while making them very easy to practice by removing social and structural barriers such as the expense or inaccessibility of masks and hand sanitizer.

## Introduction

Despite the rapid development of several vaccines against the novel SARS-CoV2 virus and its associated illness, COVID-19, a vaccinated globe remains far out of reach. Shortages in vaccine supply for low- and middle-income countries continue [1,2] while high income countries monopolize doses and widespread hesitancy persists even among those with access to the vaccine [3,4]. Meanwhile, the pandemic rages on, with 269 million cases and almost 5.3 million deaths globally towards the end of 2021 [5].

Within this context, prevention remains critical to reducing the social and economic impact of the pandemic [6]. Effective prevention behaviors (sometimes called prevention measures, barrier measures or non-pharmaceutical interventions, NPIs) against COVID-19 and similar respiratory pathogens are well-documented: mask wearing, distancing, hand hygiene, avoiding crowds, limiting non-essential travel, and quarantining after exposure [7]. However, compliance with prevention behaviors is difficult to maintain over time, and many studies have shown low or waning adherence in a variety of settings across Africa [8–11].

Understanding the factors that prevent individuals from engaging in COVID-19 prevention behaviors is necessary to the design and implementation of effective public health programs and to reduce the effects of the COVID-19 pandemic globally. Barriers to desired COVID-19 prevention behaviors are multilevel and multifaceted and operate at the individual, social, and policy levels [7]. Individual determinants include demographic factors like age, sex, and education level, knowledge of the benefits, risk perceptions, attitudes and beliefs, self-efficacy, and where people report getting information about health [11–15]. Evidence suggests a complex interplay between individual decisions and the economic, social, media, and policy environment that may hinder or support prevention behaviors, with socio-economic factors such as poverty and mobility intensely constraining the ability of the population to maintain adherence to recommended or required prevention behaviors [16–19].

The Extended Parallel Process Model (EPPM) is a useful behavioral framework that considers how people respond to a health concern, particularly based on their perceived susceptibility to the risk and the perceived severity of the consequences [20]. In the context of COVID-19, perceived susceptibility reflects individuals’ perceptions of the likelihood that they might be infected, whereas perceived severity reflects individuals’ perceptions of the likelihood that the illness would be serious for them if infected. Misinformation throughout the pandemic has directly undermined these two elements of risk perception by denying the existence of the virus, promoting the belief that certain groups of people cannot get infected, or suggesting that COVID-19 is always mild [21]. According to the EPPM, individuals who feel threatened then adopt preventative behaviors to the extent that they feel that the recommended behaviors will reduce the threat (response efficacy) and if they feel they have the resources and ability to perform them (self-efficacy) [22]. Response efficacy and self-efficacy vary based on the specific prevention behavior. For example, individuals may feel that masks do not work to prevent infection (low response efficacy) whereas physical distancing may be perceived to be useful in preventing the spread of the disease (high response efficacy). One’s perceived ability to perform the behavior, either for a short period of time or permanently, is likewise variable depending on the larger context in which the behavior is practiced and how expensive or burdensome the behavior is. People may have greater self-efficacy for hand hygiene in settings where soap and water are readily available, for instance, as opposed to their self-efficacy to limit travel in settings where employment depends on mobility.

Côte d’Ivoire, a West African setting with a moderate burden of COVID-19, is a helpful case study for exploring drivers of COVID-19 prevention behaviors. As 2021 closes, Côte d’Ivoire had 61,800 reported cases and over 700 deaths related to the SARS-CoV2 virus since the first documented case on March 11, 2020 [5]. The case load in Côte d’Ivoire has consistently been lower than Western settings yet higher than several neighboring countries. Data from a survey early in the pandemic suggested that Ivoirians had high awareness of the recommended prevention behaviors, but compliance was influenced to a greater extent by individuals’ risk perceptions, exposure to misinformation, and trust in the government’s management of the COVID-19 response [19]. However, gaps in understanding the barriers to engaging in COVID-19 prevention behaviors in Côte d’Ivoire remain. To address these gaps, a formative qualitative study was designed to explore ideational factors that may facilitate or hinder uptake of key COVID-19 prevention behaviors. The study was conducted as formative research to inform the design and implementation of social and behavior change communication activities.

## Methods

### Study design

The Breakthrough ACTION project, led by Johns Hopkins Center for Communication Programs, was tasked with addressing COVID-19 in Côte d’Ivoire through social and behavior change communication. The Breakthrough ACTION team conducted a qualitative study in Abidjan in November 2020 with funding from the United States Agency for International Development (USAID). The larger study focused on stigma related to COVID-19; the current analysis focuses on a subset of the data involving COVID-19 prevention behaviors. Findings from the study informed the COVID-19 response in Côte d’Ivoire.

### Sample

The study included in-depth interviews (IDIs) with survivors of COVID-19, individuals who had lost a family member to the disease, and health workers (such as doctors, nurses, or pharmacists). Some of the health workers had also recovered from COVID-19, and the sample included those who had directly treated COVID-19 patients and those who had not. We also conducted focus group discussions (FGDs) with members of the general population to explore social norms and community perceptions related to COVID-19. The research team selected a sample size using standard approaches for qualitative research to ensure that the sample sizes were sufficient to achieve information redundancy in the interviews and FGDs [23,24]. The sample included both men and women over the age of 18. FGDs were mixed gender, age, and educational background.

The research team used purposive sampling, an approach common in formative research to inform public health programs [25]. Participants were recruited through the national program for orphans and vulnerable children, which was the government agency in Côte d’Ivoire tasked with tracking people with COVID-19. Focal persons were identified in partnership with regional health authorities and other stakeholders and were primarily community health workers with whom the national program for orphans and vulnerable children collaborated. Focal persons recruited participants at four government-run COVID-19 treatment centers in Abidjan as well as in surrounding communities in collaboration with the Ministry of Health. They held positions of trust with communities and were given a training on the recruitment script and the nature of the study and voluntary participation. Individuals who expressed interest in participating in the study provided their contact information to the focal persons and information about their COVID-19 experiences to assign them to the appropriate IDI or FGD.

### Ethical review

The Ivoirian national research ethics committee (Comité National d’Éthique des Sciences de la Vie et de la Santé, CNESVS) approved the study, as did the Johns Hopkins Bloomberg School of Public Health Institutional Review Board [IRB#13757].

### Data collection

The study team used a written informed consent process that involved reading the information note to potential participants. Willing participants used a disinfected pen to sign consent forms prior to the FGD or IDI, and the research team kept the signed forms. COVID-19 safety procedures required by the government and the ethical review boards involved holding FGDs or IDIs outdoors or in a large room with open windows–typically at community centers or private offices. A limit of six participants per FGD enabled physical distancing. Participants and study team members were given masks and hand sanitizer. The research team verbally instructed participants to contact the study coordinator if they received a positive COVID-19 diagnosis in the two weeks following the FGD or IDI, and that request was also highlighted in the information note retained by participants. No COVID-19 cases were reported during the data collection period or in the two weeks following.

The data collection team was comprised of Ivoirian researchers with qualitative research experience and advanced degrees in sociology or demography. The principal investigator, the study coordinator, and the on-site lead researcher collaborated to facilitate a training that included research ethics as well as an orientation on the protocol and the interview guides. As part of the training, data collectors conducted a pretest with 10 individuals (2 interviews and 2 FGDs with 4 participants each), which were not included in the final sample.

The research team collected data from November 11–25, 2020. The interviews and FGDs were facilitated in French and were audio-recorded. On average, FGDs lasted one hour and fifty-nine minutes and IDIs lasted forty-three minutes. FGD guides explored attitudes and norms toward seven key COVID-19 prevention behaviors (Table 1) through a pile sorting activity that considered both response efficacy and self-efficacy around each behavior.

**Table 1:** Key COVID-19 prevention behaviors considered.

Behavior
A. Respecting a quarantine of 2 weeks after exposure
B. Keeping a distance of 1m between people
C. Wearing a mask in public
D. Washing hands frequently
E. Using hand sanitizer frequently
F. Limiting gatherings to fewer than 50 people
G. Limiting non-essential travel

In the FGDs, participants were first asked to sort behaviors based on response efficacy. According to the EPPM, response efficacy reflects whether individuals feel the behavior is effective in limiting the spread of the disease. Researchers asked the groups to categorize each behavior as “very useful,” “somewhat useful,” or “not at all useful” for preventing COVID-19. Ensuing conversations around the usefulness of the behavior allowed the research team to explore the reasons people felt each behavior was or was not effective. Participants were then asked to classify each behavior based on self-efficacy. Self-efficacy is also a central component of the EPPM, as described above, and reflects people’s confidence that they can perform the desired behavior. Researchers asked the FGD participants to sort each behavior based on whether it was “very easy to do,” “somewhat easy to do,” or “not at all easy to do.” Discussions that followed on how to categorize each behavior allowed researchers, during the analysis, to understand factors that influence people’s self-efficacy. Interviewers also asked participants to sort each behavior by perceived social norms or whether participants felt there was “a lot of pressure,” “some pressure,” or “no pressure” to observe the recommended behavior. For each sorting exercise, the interviewers probed for further insights as participants discussed and debated. Both the final consensus and any dissenting opinions were noted. The interviewer then further explored attitudes and social norms by asking what people thought of those who practiced these behaviors. Interviewers did not conduct the pile sorting activity in IDIs with COVID-19 survivors or those who had lost family members to COVID-19, but rather explored individuals’ personal experiences with COVID-19 from diagnosis and treatment through recovery and return to the community. The IDI guide for health workers elicited insights on barriers and facilitators to preventive behaviors as well as overall perceptions and experiences treating COVID-19. As this analysis focuses on the seven key prevention behaviors outlined in Table 1, we draw primarily from the FGD data, which are then complemented by insights gathered from interviews with COVID-19 survivors and health workers as appropriate.

The research team transcribed all IDIs and FGDs word-for-word in French. A member of the research team validated all transcripts by spot checking five minutes of audio to the transcription in three places for each transcript. The transcript was sent back for a full review if any errors were found, and the process repeated until the transcripts accurately mirrored the audio.

### Analysis

The research team conducted a preliminary thematic analysis of the data through a participatory data analysis workshop [26,27]. A group of 14 stakeholders, including staff from the Breakthrough ACTION programmatic team, the study team including data collectors, and representatives from the Ivoirian government spent five days reading transcripts and discussing insights in small groups. Insights were also entered into a matrix that documented the key point and illustrative quotations. In plenary sessions, each small group shared insights that were then collaboratively synthesized into themes with illustrative quotations. These inductive themes–along with deductive topics from the interview guides including those informed by the EPPM such as response efficacy, self-efficacy, perceived susceptibility, and perceived severity of COVID-19 –comprised the codebook. Following the analysis workshop, the data collectors coded all transcripts in Atlas.ti using the codebook. They double coded 19% of transcripts and met to discuss and resolve discrepancies. The coded data were then extracted for each behavior and assigned to one co-author (seven co-authors participated: the four researchers who conducted the interviews, the lead consultant, and the first and last authors). Each co-author read the data for his or her assigned behavior and completed a template that summarized the key points and illustrative quotations. Co-authors met to debrief the analysis and identify cross-cutting themes. An iterative process of writing the findings and providing feedback to ensure that the summary accurately reflected the results allowed for credibility and reflexivity checks, with the final themes and illustrative quotations approved by all co-authors.

### Inclusivity in global research

Additional information regarding the ethical, cultural, and scientific considerations specific to inclusivity in global research is included in the Supporting Information (S1 Questionnaire).

Overall, 156 individuals participated in this study (Table 2). There were 29 IDIs, 17 of which were with health workers, and 24 FGDs (with 127 total FGD participants). There were more men (n = 89) than women (n = 67) in the final sample. The research team noted the sex of all participants, but in some cases, the participant preferred not to share their age which the research team respected.

**Table 2:** Study participants.

Research approach	Participant type	Total		
Focus Group Discussions	*General population*			
	Knew someone with COVID-19	12 groups (24 females, 36 males)		
	Did not know someone with COVID-19	12 groups (32 females, 35 males)		
Individual interviews	*General population*	**Female**	**Male**	**Total**
	Recovered from COVID-19	4	4	8
	Lost a family member to COVID-19	1	3	4
			
	*Health workers*			
	Did not directly treat COVID-19 patients	3	8	11
	Directly treated COVID-19 patients	3	3	6

In the following sections, we examine perceptions regarding seven COVID-19 prevention behaviors through an exploration of two key elements of the EPPM–namely, participants’ response efficacy and self-efficacy to perform the desired behaviors–as well as perceived social norms. While these constructs cut across the behaviors, we analyze each behavior in turn, as the nuances specific to each behavior are important to consider as health authorities make decisions about which restrictions to maintain and how to encourage widespread adoption.

## Results

Participants described low uptake of the seven COVID-19 prevention behaviors, while expressing a strong belief in the usefulness of certain behaviors for preventing infection. Even for behaviors considered useful (response efficacy), low self-efficacy hindered the ability of individuals to adopt or sustain the behaviors, particularly after the initial phase of the pandemic. Rumors questioning the existence of COVID-19 in Côte d’Ivoire or touting African immunity trivialized the disease and related prevention behaviors. Meanwhile, unfavorable norms reinforced the perception that behaviors were unnecessary or impractical. Furthermore, participants framed their resistance to many of the behaviors in term of social cohesion, feeling that masks, distancing, limiting travel, quarantine, and even handwashing were anti-social and undermined interpersonal relationships. Table 3 provides illustrative quotations for each prevention behavior by determinant. In the following sections, we describe general perceptions toward each behavior in turn. We give particular attention to self-efficacy, response efficacy, and perceived social norms, as these constructs framed the design of the study and our interpretation of the responses.

**Table 3:** Illustrative quotations by determinant for key COVID-19 prevention behaviors.

Illustrative quotations
**A. Respecting a quarantine of 2 weeks after exposure**
*Self-efficacy* It is very difficult for someone who has a job and is responsible for a family, a little family, to quarantine himself. It becomes even more difficult given his family situation, his children. How will his wife, his children eat? While in quarantine, how can he meet his family’s needs? (male, 40–49 years old, FGD, knew someone who had COVID-19)
*Response efficacy* Maybe it is because of life’s challenges that some people go out, as she said so well, to get their daily bread. Otherwise, they know that quarantine is important to avoid the spread of COVID. (male, 30–49 years old, FGD, knew someone who had COVID-19)
*Perceived norms* For people to go into quarantine, the community needs proof, they need to see people sick with COVID so that they can believe, but once it is confirmed that the person has COVID, the community itself will push quarantine so as not to be exposed to the disease. (male, 40–49 years old, FGD, knew someone who had COVID-19)
**B. Keeping a distance of 1m between people**
*Self-efficacy* In the beginning we believed that it [keeping a one-meter distance] could help us. We tried to do it, but then when we saw, I’m referring to the election again, when we saw all the people that were in the stadiums and we saw that there wasn’t even a centimeter, so we, too, lost interest in that. So, there is not one meter. Here, I love my brother and I approach him. I tell myself that, in fact, we’re all going to die [of something]. In Europe the disease exists, but in Côte d’Ivoire, frankly, we don’t believe in it. (female, 20–29 years old, FGD, did not know someone who had COVID-19)
*Response efficacy* Some believe, but others question it and, at the same time, they shake hands, saying that it isn’t for us, it is the disease of the whites, it doesn’t affect us, it doesn’t kill blacks, so as a result, they don’t maintain a distance of 1 meter. (female, 20–29 years old, IDI, health provider who cared for COVID-19 patients)
*Perceived norms* For me, I think that it isn’t in our customs, we aren’t used to keeping 1 meter distance. We’re used to coming together to work, sitting in the same place, even for eating, we can sit side by side, so it’s not ingrained in us, in our customs, so it is very difficult for us to respect the 1 meter. (male, 20–29 years old, FGD, knew someone who had COVID-19)
**C. Wearing a mask in public**
*Self-efficacy* With respect to our climate in Africa, it is already hot. So, if you have to wear a face mask and you aren’t in a vehicle, if you have to walk in the sun, you’re hot, you have the impression that you’re suffocating. That’s why some people can’t stand [it]. (male, 30–39 years old, FGD, knew someone who had COVID-19)
*Response efficacy* Some people even say that your disease is in the face masks. That’s why I don’t wear them. (female, 30–39 years old, FGD, knew someone who had COVID)
*Perceived norms* This discouragement is affecting more and more providers in health facilities, all providers in health facilities. Some, so as not to be poorly viewed, knowing the consequences, feel obligated to remove their masks so as not to appear ridiculous in the community. (female, 30–39 years old, IDI, health provider who cared for COVID-19 patients)
**D. Washing hands frequently**
*Self-efficacy* [Handwashing with soap] can be a little difficult. The first challenge is accessibility to a water point. It isn’t everywhere that we have running water and, in addition to that, we find it a bit restrictive to look for water and then soap to wash our hands. (male, 50–59 years old, IDI, health provider who did not care for COVID-19 patients)
*Response efficacy* People do it. They think that it can protect them, but it is a question of how often, because you wash your hands, but it isn’t everywhere that you have the ability to wash your hands. (male, 50–59 years old, IDI, health provider who did not care for COVID-19 patients)
*Perceived norms* In my bar, there are buckets, so when people come, you don’t have to tell them, they see the soap, they will wash their hands without you telling them. It is our habit. The African, you don’t even need to tell him, he knows, he washes his hands. (male, 40–49 years old, FGD, did not know someone who had COVID-19)
**E. Using hand sanitizer frequently**
*Self-efficacy* At the beginning we had little bottles of hand sanitizer that cost three hundred, five hundred, and in record time they increased to one thousand francs. And not everyone could afford it. That’s what made people lose interest. Otherwise, if I’m asked to choose between the use of water and hand sanitizer, I prefer hand sanitizer over soap and water because you can carry it everywhere very easily. (male, 40–49 years old, FGD, did not know someone who had COVID-19)
*Response efficacy* They find it [hand sanitizer] useful because they think of disinfecting their hands… it kills even more bacteria. Even if they don’t believe in COVID, they know that it kills more bacteria in their hands. (male, 40–49 years old, FGD, knew someone who had COVID-19)
*Perceived norms* One day I went with my daughter to Yopougon. In the shared taxi, I saw a child touch the chair. So, I put a little [hand sanitizer] in his hand. There were two girls in front who started to make fun of me. (female, 30–39 years old, FGD, did not know someone who had COVID-19)
**F. Limiting gatherings to fewer than 50 people**
*Self-efficacy* [Limiting gatherings to 50 people] wasn’t easy to respect because it also depends on the context. For example, in a bar, you know that it is a question of money, the more people, the greater the profit. So, for him, it will be hard to limit to 50 people if he could have more. So, it isn’t just about disease prevention; there is that other [economic] part too that we can’t forget. (male, 40–49 years old, IDI, health provider who cared for COVID-19 patients)
*Response efficacy* Limiting gatherings to 50 people can slow the spread of the virus in the sense that, with more that 50 people, it is difficult to social distance and, the more people who gather, the easier it is to catch this disease. The less we gather, the more difficult it is to spread or to catch this disease because social distancing will be respected. That’s what I think it’s about. (male, age not provided, FGD, did not know someone who had COVID-19)
*Perceived norms* Everyone wants to attend… and if we set a certain limit on people, other people don’t respect it. Some people give invitation cards, but, despite that, there are others who come and of course they want to see inside, see, eat, and be comfortable there too. That’s what makes them not respect [the 50-person limit on gatherings]. (male, 20–29 years old, FGD, knew someone who had COVID-19)
**G. Limiting non-essential travel**
*Self-efficacy* You’ll be obligated to go out. For example, if you work, you’ll be obligated to go out. You won’t say that you’ll respect it and stay at home because that is what provides your food, you can’t sit at home. And you’ll say no, even though they say not to go out because of corona, you’ll lose your job. (male, 20–29 years old, FGD, did not know someone who had COVID-19)
*Response efficacy* Now, everyone is moving all the time. We always have something to do, travel, move, so now, no, it is no longer useful. Before, yes. (male, 50–59 years old, FGD, did not know someone who had COVID-19)
*Perceived norms* The African is someone who is essentially free in his head. The ability to visit friends and relatives is essential. They can’t understand that because of a disease they can’t visit loved ones. It’s difficult to get through. (male, 50–59 years old, IDI, health provider who did not care for COVID-19 patients)

### A. Respecting a quarantine of 2 weeks after exposure

Participants rated quarantine for COVID-19 patients as a very effective behavior and understood the importance of preventing further transmission but emphasized the economic and social difficulties.

#### Response-efficacy

Support for self-imposed isolation due to potential exposure was virtually non-existent, but, in general, participants agreed that someone who has COVID-19, as confirmed by a positive test result, is morally obligated to isolate themselves from others to prevent the spread of the virus. People who questioned the usefulness of quarantine, also questioned the validity of the COVID-19 test, asking what proves that the person has COVID-19. This lack of trust in the COVID-19 test, reflects a broader distrust in the government when it comes to COVID-19 and a sentiment that the matter of quarantine was poorly handled by the government in Côte d’Ivoire. Across numerous FGDs and IDIs, participants recounted that, at the beginning of the pandemic, celebrities (politicians, artists, athletes) and relatives of government officials returned to Côte d’Ivoire from abroad, refused to respect the government-imposed central quarantine and were allowed to quarantine at home. This favoritism undermined the legitimacy of the pandemic in Côte d’Ivoire and contributed to a pervasive fear of quarantine in government facilities. Participants equated quarantine to a “death sentence” or even a “slaughtering house,” and shared the general perception that COVID-19 patients in government facilities were not well cared-for. Fear of forced quarantine paired with fear of stigma also led to a fear of testing and avoidance of hospitals. As one FGD participant said, “*If a person suspects they have the disease*, *because of the eyes of others [stigma] and fear*, *they will say if I go there*, *they will retain me and put me in quarantine*. *So that person starts to treat themselves by taking indigenous remedies*” (female, 40–49 years old, FGD, did not know someone who had COVID-19).

#### Self-efficacy

Quarantine was viewed as an economically and psychologically difficult sacrifice for the greater good. Participants felt that, in absence of support from the government, quarantine for two-weeks was only feasible for people with economic means. Most Ivoirians, who earn their living day-to-day, neither have the space in their home to quarantine, nor the ability to meet their daily needs for food, water, and rent, during a two-week quarantine. Participants voiced the need for government support for sufferers of COVID-19 and their families, in the form of humane government quarantine facilities and compensation for lost income during quarantine. This government support was largely felt to be completely lacking or misappropriated. Participants also spoke of the psychological toll of quarantine, describing Ivorians as people who value freedom, mobility, and crave social interaction. A health worker said, “*Quarantine*, *in the mentality of most people*, *is a prison*, *a deprivation of liberty that is difficult to accept*” (male, 50–59 years old, IDI, health provider who did not care for COVID-19 patients). A FGD participant said, “*I would say very very difficult*. *As I said before*, *every man aspires to move freely*. *Staying in quarantine is very difficult*” (male, 30–39 years old, FGD, did not know someone who had COVID-19). Overall, people who did self-isolate if sick were viewed positively, as sacrificing themselves for others. However, the word “quarantine” was tainted by the negative perceptions of quarantine in government facilities, in contrast to self-isolation at home which wasn’t labeled quarantine by some, and by stigma. Quarantining, either at a government facility or at home, was felt by some to betray a COVID-19 positive status that would leave a permanent mark on the family. Participants expressed an internal, moral pressure to quarantine if a person knows they are sick and an external pressure to quarantine if others know you are sick. Participants who had never met anyone with COVID-19 or who doubted its existence abstained from questions around normative pressure to quarantine, having not experienced contact with the disease first-hand.

#### Social norms

In Côte d’Ivoire, isolating a sick person is to stigmatize them and goes against the Ivoirian culture. As one health worker explained, “*In Côte d’Ivoire*, *and in Africa in general*, *[quarantine] is the measure that is the most difficult for the population to accept…*. *It isn’t in the culture*. *If you are sick*, *you have family around you*. *Isolating people like that is the measure that will derail the entire system because*, *in our culture*, *if you quarantine someone*, *you stigmatize them*” (male, 50–59 years old, IDI, health provider who did not care for COVID-19 patients). Positive deviants among the participants expressed that it is possible to isolate a sick family member at home while respecting all safety measures and still caring for them, not abandoning them. A FGD participant said, “*If my family were contaminated*, *I would follow the measures listed here*, *but I wouldn’t abandon the person*. *It isn’t humane*” (female, 30–39 years old, FGD, did not know someone who had COVID-19). Another FGD participant said, “*If a brother or someone I know is sick*, *we’ll adopt the barrier measures*. *He will be quarantined in a room*, *and we’ll give him the necessary love*” (female, 20–29 years old, FGD, did not know someone who had COVID-19).

### B. Keeping a distance of 1m between people

Despite the documented importance of physical distancing in the fight against COVID-19, there were differing perceptions of its usefulness among study participants. This ambivalence was reflected in the respondents’ practical assessment of the effectiveness of physical distancing in relation to barriers in the social context.

#### Response-efficacy

Most participants understood how physical distancing can limit the spread of the virus; however, successful application of distancing was thwarted by a strong desire for social cohesion. People valued distancing as an alternative to avoiding crowds, as one FGD participant said, “*I think that when there are ceremonies and marriages and things that it is useful to maintain distance because there are more than 50 or 100 people*, *more than 200 people*. *So I think it is good*” (male, 40–49 years old, FGD, did not know someone who had COVID-19). Distancing was not framed in terms of usefulness or uselessness, but rather in the context of social dynamics and mobility that punctuate people’s daily lives. In other words, people considered whether the behavior is “useful” less from a medical perspective and more from the angle of the social proximity in which travel and valued interpersonal interactions take place. Also, this behavior was perceived as non-useful and trivialized for those whose interpretation of the pandemic was influenced by misinformation, as exemplified by a participant who said:

*People who insist on the one meter are stupid because in the first place we’ve told you that it doesn’t exist*, *you’ve never seen it*, *because us Africans*, *we like to see to believe*, *so something that I’ve never seen*, *that I’ve never even seen*, *and around me*, *no one has it*, *my head isn’t in it*, *so I can’t worry about one meter*. *And now you apply it*, *I already find you bizarre*, *what are you looking for*? *Because there is nothing*. *Corona is for the whites*, *it’s for the rich*, *we haven’t had it yet*, *so it isn’t in our heads to say one meter like that*. (male, 20–29 years old, FGD, did not know someone who had COVID-19)

Other participants similarly described the belief that Africans are more resistant to COVID-19, that the virus has a mystical origin, or that COVID-19 is over in Côte d’Ivoire.

#### Self-efficacy

Physical distancing confronts the norms that govern social interactions in Côte d’Ivoire. Gestures of familiarity such as greetings, handshakes, and hugs reinforce social bonds. Distancing, by definition, sets one apart from others which goes against the very grain of Ivorian culture. Keeping 1 meter from others was perceived as more feasible among strangers than among friends and family, as one participant described, “*People think that they need to keep a distance from people that aren’t from the family*, *that aren’t from the neighborhood*. *So*, *if you go to a supermarket*, *you know it’s a measure*, *so you keep your distance*. *Because you don’t know them*, *so there it is easy*, *but in the community*, *in the neighborhood*, *in the house*, *it isn’t easy*” (female, 30–39 years, FGD, knew someone who had COVID-19). In some cases, people who insisted on social distancing among familiar others were mocked as overly compliant “puppets” of the government.

In addition, socio-economic factors contributed to an inability to maintain physical distance between individuals. Indeed, housing and travel patterns (overcrowded housing and public transport) limit the feasibility of this behavior. Difficulty in complying with distancing was more marked in spaces with intense and "uncontrolled" interactions such as markets and public transportation, in contrast with other settings such as places of worship, pharmacies, supermarkets, and banks, where distancing was often imposed. The inability to systematically maintain 1 meter from others led some participants to prioritize other behaviors such as handwashing, use of hand sanitizer, or mask wearing to the detriment of respecting physical distancing. On the other hand, some participants felt keeping 1m distance was easier than the other behaviors because it did not require purchasing anything, such as soap, sanitizer, or masks.

#### Social norms

Perceived social norms were unfavorable to physical distancing and reflected the government’s failure to model recommended COVID-19 prevention behaviors by holding political rallies and televised funeral rites for a deceased minister. Reported lack of compliance with distancing in health centers further undermined normative support for this behavior. These actions were seen as contradictions by study participants or even as reinforcing misinformation that questioned the existence of the virus.

### C. Wearing a mask in public

Participants were divided on whether masks were useful, but most people felt masks were not easy to wear routinely or long term given the cost and hot climate.

#### Response-efficacy

There was considerable variation in beliefs about whether masks are helpful, neutral, or harmful. Some thought that masks were contaminated with the SARS-CoV2 virus and would infect people. The suspicion about masks intersected with stigma around the origin of the illness, as described by a participant who said that most masks “*come from China and the disease started there*. *The Chinese want to propagate [the disease] here*, *so one thought is that it is in the masks*” (female, 30–39 years old, FGD, knew someone who had COVID). On the other hand, some people did not feel the masks were useful or necessary because they did not believe in COVID-19 at all or that COVID-19 was still in Côte d’Ivoire. A participant said, “*they are fed-up*. *When you say*, *‘you have to wear a face mask*,*’ insults follow*: *‘You are pissing people off with your Corona business… there is no Corona here’*” (female, age not provided, IDI, recovered from COVID-19). As with other preventive behaviors, this doubt about the existence of the virus undermined compliance with mask wearing. Health workers, on the other hand, valued mask wearing as a way of protecting others. One health worker explained, “*If you love your neighbor*, *it means that you also love yourself*. *So*, *you have to wear your mask correctly*” (female, 30–39 years old, IDI, health provider who cared for COVID-19 patients). Participants in the general population often framed the need for a mask around whether they were around people close others or strangers. One individual differentiated between the home neighborhood as opposed to the larger city, saying “*Here*, *in the neighborhood*, *when we don’t go out*, *we’re just here in our sector*, *we find that it’s not useful [mask wearing]*. *But when we want to go out*, *it is imposed on us*” (female, 30–39 years old, FGD, did not know someone who had COVID-19). The only valid time to wear a mask in the neighborhood was if you were diagnosed with COVID-19, a belief that led to harmful assumptions about people who did wear masks. One participant described being afraid of people wearing a mask, saying, “*someone who always has their mask on*, *we fear him*, *because we think he has the disease*” (male, 30–39 years old, FGD, knew someone who had COVID-19). Another participant described being temporarily expelled from her housing situation because wearing a mask exposed her COVID-19-positive status.

#### Self-efficacy

Wearing a mask was difficult for community members to respect. According to participants in FGDs as well as health workers, the main issues were comfort and cost. Participants described people objecting that masks “*suffocate them*. *So*, *some people don’t wear them*. *Not because they don’t know its importance*! *But some people say that it makes them tired*, *so that’s why people have let up [on mask wearing]*.” (female, 30–39 years old, FGD, knew someone who had COVID-19). Particularly in the heat, masks were considered untenable. As with other barrier measures that had to be purchased, the cost was prohibitive for some participants.

#### Social norms

Mask wearing, except in places and spaces where it was required, was considered unacceptable for most people after the initial phase of the pandemic. Wearing a mask was seen as setting oneself apart, as described by a participant who said, “*we find him superior to us*, *because he thinks his Corona*, *his COVID-19*, *is so important*” (female, 20–29 years old, FGD, knew someone who had COVID-19). COVID-19 survivors were more determined to wear masks; their experience with the illness gave them motivation to overcome unfavorable social norms. One participant who had survived COVID-19 said that, after having COVID-19, he continued to wear a mask even when others did not, saying, “*with respect to the barrier measures*, *I was the first to wear a mask*” (male, age not provided, IDI, recovered from COVID-19). Another participant who had recovered from COVID-19 perceived her risk to be higher, saying she was now convinced COVID-19 exists and “*in my opinion*, *the only way to avoid the disease is to wear a mask*.” (female, age not provided, IDI, recovered from COVID-19).

People described that wearing masks in public places was acceptable where it was required. Mandates and enforcement appeared to have a favorable impact on mask uptake in crowded settings, as a participant explained, “*In my church*, *there are people who come without a face mask*, *but it is mandatory*, *without a face mask*, *you can’t enter*. *So*, *they are obligated to wear it*. *If it were just up to them*, *they would enter without a face mask*” (female, 20–29 years old, FGD, did not know someone who had COVID-19).

### D. Washing hands frequently

The behavior of handwashing benefited from prior promotion. Perceived usefulness was undermined by misinformation (rumors), and people felt that the behavior was somewhat inconvenient to practice.

#### Response-efficacy

Among participants who felt that COVID-19 was both real and severe, handwashing was valued as a way to protect oneself. Those who questioned the efficacy of handwashing often also questioned the existence of COVID-19 in Côte d’Ivoire and subscribed to a narrative of African invulnerability to the disease:

*In the beginning*, *there was really a scare everywhere*. *But after that*, *people in families*, *in neighborhoods*, *people don’t pay much attention anymore*. *Me*, *for example*, *at my work there was a time when everyone washed their hands*. *The buckets of water are still there today*, *but it is rare that people wash their hands because*, *by the grace of God*, *God is with us*, *when you look at Africa*, *if it isn’t the white*, *yellow or other skin*, *in Africa*, *really*, *blacks don’t die like that*. *Even the second wave that is disfiguring Europe*, *America*, *in Africa we don’t hear that*.” (male, 50–59 years old, FGD, did not know someone who had COVID-19).

Other participants felt that handwashing was the only behavior worth continuing in light of waning risk perception, as described by one individual who lost a family member to COVID-19, “*I don’t care*. *It is finished*. *I don’t care*… *what will happen*, *will happen*. *I’m careful to a certain extent*, *I wash my hands*, *but I’m no longer like I was before*, *at the beginning*, *when we barricaded our noses*, *we didn’t visit*, *we didn’t touch people*. *Listen*, *I go where I want*” (male, age not provided, IDI, lost a family member to COVID-19). This fatalism regarding infection was common among people who felt both low risk perception as well as low self-efficacy to maintain the prevention behaviors.

#### Self-efficacy

The installation of handwashing stations at the entrances to communities, businesses, and homes temporarily boosted self-efficacy and favorable perceived social norms at the beginning of the pandemic. However, self-efficacy eroded over the course of the pandemic as people felt unable to maintain the initial systematic handwashing over the long term and began to feel impervious to the disease, as described by a participant, “*At the beginning*, *we washed our hands a lot*. *Every time we went out*, *we washed our hands*. *In every courtyard*, *even*, *some people even put buckets and things*, *but as we went along*, *we started to tend to live a little bit with the disease”* (male, 20–29 years old, FGD, knew someone who had COVID-19). As the pandemic progressed, fear waned as did compliance with the prevention behaviors.

#### Social norms

Handwashing benefitted from prior normative support, as a practice that predates COVID-19 and has long been promoted to fight infections. In that regard, handwashing escaped the stigma attached to other prevention behaviors that emerged specifically in response to COVID-19. Promotion in schools and through various disease-based programming has led to general acceptability of handwashing as a preventative practice. However, the acceptability of handwashing is often limited to specific times or rituals (like before meals). A health worker explained that the inappropriate timing of handwashing can be perceived as insulting: “*very few people will wash their hands*. *Imagine that you shake my hand and that you immediately go and wash your hands*. *What impression would I have of the fact that after shaking my hand*, *you go and wash them*? *That’s not accepted in our culture*” (male, 50–59 years old, IDI, health provider who did not care for COVID-19 patients). Across groups with respect to public places, participants characterized the existence of handwashing stations as a form of pressure as well as being asked to wash hands before entering a places like supermarkets. At the household level, participants who knew someone who had had COVID-19 appeared to feel and exert more pressure within families to wash hands regularly.

### E. Using hand sanitizer frequently

The convenient and pro-social nature of hand sanitizer was offset by the cost and inaccessibility at certain points during the pandemic.

#### Response-efficacy

Participants generally expressed the belief that using hand sanitizer not only prevents COVID-19 but is also effective against other diseases that can be transmitted by dirty hands. However, some participants from the general population, especially those who did not know someone who had had COVID-19 and/or who did not believe in the disease, did not believe that hand sanitizer was useful in preventing COVID-19.

#### Self-efficacy

Frequent use of hand sanitizer was perceived by study participants to be the easiest COVID-19 prevention behavior to adopt and maintain over time. They opted for hand sanitizer as a way of foregoing other, more challenging, behaviors to adopt, as described by a participant who said, “*One of my friends was telling me that when he wears the mask*, *it suffocates him*. *So everywhere he goes he takes hand sanitizer*. *Every time he touches something*, *he puts a little in his hands*” (male, 20–29 years old, FGD, knew someone who had COVID-19). Given that cost (and stock outs at the beginning of the pandemic) were the primary barriers to frequent use of hand sanitizer, some participants advocated for making hand sanitizer available to the population free of charge. Hand sanitizer is relatively low cost, quick and fun to use, portable, discrete, and therefore inoffensive, and easy to share making it a prevention behavior that contributes to social cohesion and is culturally acceptable. A health worker described this link between self-efficacy and social cohesion, saying, “*If you have your little hand sanitizer*, *that hand sanitizer*, *it can be shared*, *you see*? *You can share it*, *so it is easy for them to have*” (female, 20–29 years old, IDI, health provider who cared for COVID-19 patients).

#### Social norms

Frequent use of hand sanitizer as a social norm ebbed and flowed with risk perception related to highly mediatized public health threats. Participants recounted that hand sanitizer was made available in most public places during the Ebola crisis in 2014 and then disappeared when the perceived threat of Ebola was gone, and a similar pattern was emerging with COVID-19. The availability of hand sanitizer and reminders to use it before entering public places (banks, pharmacies, hospitals, places of worship, etc.) generated a positive social norm around the frequent use of hand sanitizer and boosted self-efficacy by overcoming barriers related to access.

### F. Limiting gatherings to fewer than 50 people

Both avoiding large gatherings and choosing to limit one’s own gatherings to fewer than 50 people were felt to be useless and difficult, both because people did not understand the benefit and large gatherings are part of the fabric of Ivoirian culture.

#### Response-efficacy

Most study participants were skeptical of the response-efficacy of limiting gatherings to 50 people. Participants questioned the scientific basis for the 50-person threshold and felt that limiting gatherings to 50 people would not guarantee protection against COVID-19. Changing guidance about the number of people who could safely gather and large public events undermined trust in the efficacy of this recommendation, as described by an individual who said, “*at first it was 50 people but now they’re saying it’s 200 people*. *You see*, *I think that even here*, *I think that the State itself thinks that it is not important*” (male, 20–29 years old, FGD, knew someone who had COVID-19).

#### Self-efficacy

Self-efficacy related to avoiding large gatherings or limiting gatherings to fewer than 50 people was low, given socioeconomic and cultural constraints. Participants expressed being unable to avoid crowded places like markets and public transportation. Institutions such as administrative offices, banks, pharmacies and places of worship were able to impose restrictions on the number of people, but individuals expressed being unable to ban family members and friends from events they would have otherwise attended.

#### Social norms

Social norms favor large gatherings for the celebration of important life events as opportunities to express social solidarity, strengthen social ties and show hospitality. Participants described others who tried to limit the size of social gatherings as “mean,” “selfish,” “cheap,” and “antisocial.” One participant described it as “*not easy at all because the men*, *the people in the community are called to live together*, *every day*, *they have to share their problems*, *their worries*, *their emotions together*. *Forcing them to respect the fifty people*, *the fifty people in a gathering*, *I think that it is impossible*, *it is difficult*” (male, 30–39 years old, FGD, knew someone who had COVID-19).

### G. Limiting non-essential travel

Limiting travel was a complex behavior as participants weighed the perceived importance of the prevention behavior against the duration of lockdown.

#### Response-efficacy

Participants understood why limiting movement would limit the spread of the virus, but the term “non-essential” created confusion because most travel was deemed essential for economic or sociocultural reasons. Participants described maintaining certain behaviors while allowing themselves to travel, “*they don’t find it useful*, *they say to themselves that they can travel*, *they can move around*, *there is no problem*. *What people have retained in this story of Coronavirus*, *what they have retained to fight disease is masks*, *hand sanitizer*. *As for traveling*, *I don’t think it is important*, *he moves about*. *If he’s in a car and he wears a mask that is enough*” (male, 30–39 years old, FGD, knew someone who had COVID-19). This tendency to observe certain prevention behaviors and not others was common and often influenced by economic realities.

#### Self-efficacy

Participants felt they could postpone certain travel, yet family obligations were particularly difficult to avoid, as described by a health worker who said, “*maybe for 1 month you can limit [travel]*. *Beyond that*, *there are difficulties*, *for example*, *there’s a funeral*, *the guys have to go to the village”* (male, 50–59 years old, IDI, health provider who did not care for COVID-19 patients). The ease or difficulty of complying with this behavior also depends on social status and type of work. Many participants expressed that it would be easy for well-to-do families (with stored resources to meet their needs for a longer period of time) to limit their travel, and more difficult for those who are less well off or are living hand-to-mouth. Receiving material assistance and food supply facilitated adopting this behavior, whereas misinformation–particularly skepticism that COVID-19 exists–undermined compliance. As limited travel disrupts social habits that involve mobility, contact and interaction with others, participants expressed considerable skepticism that the behavior could be maintained long term.

#### Social norms

Participants felt that members of the general population did not limit travel and pointed to a lack of modeling on the part of public figures. Within communities, there was little or no pressure from peers to limit non-essential travel, both due to a general disbelief in COVID-19 and a shared understanding that socioeconomic priorities came before observing ambiguous government restrictions. People who encouraged others to limit travel were perceived as "wimpy," "lazy," "overzealous," or "rich”. Notably, those who felt the behavior was important tended to have some personal exposure to COVID-19 through their family or social networks.

## Discussion

In this qualitative study, participants exhibited moderate response efficacy and low self-efficacy for the majority of recommended behaviors. Self-efficacy was particularly limited by economic realities–the inability to afford products like masks and hand sanitizer or the need to travel and interact with others to earn a living inhibited physical distancing or quarantining. With the exception of hand hygiene, social norms were highly unfavorable to the prevention behaviors, in part because of a lack of modeling by public figures and pervasive misinformation and conspiracy theories, such as beliefs that questioned the existence of COVID-19, insisted that Africans were immune, or promoted alternative prevention methods and therapies. The influence of social cohesion on each behavior was striking–behaviors that undermined interpersonal connectedness were perceived to be unacceptable or impossible. Taken together, these findings are consistent with other studies that suggest low risk perception of the disease, cultural norms, and misinformation reduced adherence with recommended COVID-19 prevention behaviors [18,28,29].

Overall, people felt the prevention behaviors were very or somewhat useful. Participants close to someone who had survived or died from the virus tended to express higher response efficacy. Except for limiting gatherings to 50 people and the problematic term “non-essential” travel, participants largely understood how the other COVID-19 prevention behaviors could help limit the spread of the virus. Consistent with other studies in the region, a belief that certain prevention behaviors are necessary to control the pandemic appeared to influence individual compliance [30]. However, a failure to model prevention behaviors by influential figures led to a perceived double standard and doubt in the efficacy of the recommended behaviors. Relatedly, widespread disbelief in the existence of COVID-19 in Côte d’Ivoire (stemming from a lack of trust in the government and a need to “see it to believe it”) and narratives of African immunity to COVID-19 undermined response-efficacy. If there is no threat, there is no need for a response. Participants who knew someone who had had COVID-19 were less likely to subscribe to such narratives and expressed greater response-efficacy.

Self-efficacy is inextricably intertwined with cultural acceptability and socioeconomic factors. The prevention behaviors that are not culturally acceptable, most often because they are perceived as creating social distance, also suffered from low self-efficacy because people did not feel they could sustain behaviors that others may find offensive. In addition, almost all the prevention behaviors had barriers related to socioeconomic status and participants delineated the differences between people who had the luxury of following the prevention behaviors because they had a comfortable and spacious place to live and all their needs were met without having to leave the house and people who needed to leave the house on a daily basis or they and their families would not eat or be able to pay their rent. Furthermore, people often felt that the prevention behaviors were feasible for a limited period (high “acute” self-efficacy), but that feasibility deteriorated as the pandemic lingered (low “durable” self-efficacy). Participants expressed high compliance with the recommended prevention behaviors at the beginning of the pandemic when everyone was afraid, but low compliance at the time of data collection, ten months later. This drop-off in compliance is consistent with findings reported elsewhere in the region [8,11].

Participants in the study described social norms that were largely unsupportive of COVID-19 prevention behaviors and particularly unsupportive of behaviors that bucked cultural norms around greetings, hospitality, and care for the sick. For a short time at the beginning of the pandemic, social norms stretched to accommodate more frequent handwashing, frequent use of hand sanitizer, and mask wearing. As perceived risk waned, social norms returned to pre-pandemic standards and the aforementioned prevention behaviors were relegated to places and spaces where they were required (for example wearing a mask in public transportation or washing hands before entering a pharmacy). Stigma related to COVID-19 further undermined normative support for the prevention behaviors as the behaviors became associated either with being ill (for example, a person who quarantines for 14 days or wears a mask when not required must have COVID-19) or with negative stereotypes of people who embrace the prevention behaviors as fearful, gullible, or stupid. Prevention behaviors that predate COVID-19, like handwashing, or that were perceived to protect against a broad array of diseases, like use of hand sanitizer, escaped stigma to a certain extent. More novel behaviors such as masks were less acceptable.

The drive for social cohesion permeates every aspect of Ivoirian culture in the minds of study participants. Any prevention behavior that could be perceived as creating physical or social distance between oneself and others threatened social cohesion. At the beginning of the pandemic, people could comply with the prevention behaviors without being labeled “anti-social” because everyone was afraid, and the temporary norm was to comply. In this sense, during the acute phase of the pandemic, the prevention behaviors aligned with dimensions of social cohesion such as belonging and participation, the sense that we are facing this threat together and a willingness to participate in a shared response [31]. But soon thereafter, misinformation and denialism undermined a framing of the prevention behaviors as pro-social, and instead adherence to recommended behaviors was considered anti-social, either by reinforcing existing inequities and social divisions along class lines, or simply interfering with interpersonal relationships. As the norm shifted back to “business as usual,” compliance with the prevention behaviors became less culturally and socially acceptable and more stigmatized and stigmatizing. As COVID-19 was seen to be a disease of others, a person who practices COVID-19 prevention behaviors is guilty of “othering” those around them and thereby distances, isolates and stigmatizes themselves, in keeping with insights from other settings [17,32]. A non-judgmental narrative that those who practice the prevention behaviors are simply “protecting oneself and others from disease” was largely absent from the general discourse by the time data were collected. Only use of hand sanitizer and quarantining oneself for 14 days escaped “anti-social” labels, although quarantine was not free from stigma as a person who quarantines is assumed to have COVID-19 (self-imposed quarantine due to possible exposure was virtually non-existent among study participants).

The prevention behaviors intersected and promoted or hindered one another. Certain behaviors are considered useful only as they facilitate other behaviors; for example, limiting gatherings to 50 people is only useful in allowing people to maintain 1m distance. Similarly, participants described selecting which behaviors to follow, hoping that the behaviors they found easier to implement would be sufficiently protective.

In terms of limitations of the study, the focus groups were mixed on age and gender, and some participants expressed discomfort sharing their age, which limited our ability to analyze the data by age and gender. The study also took place in an urban setting and did not account for viewpoints from rural participants. Future studies could complement these findings by conducting FGDs with homogeneity by gender, age, and urban or rural setting to facilitate comparisons across these important social locations.

## Conclusion

The findings from the study described here informed the social and behavior change strategies adopted by the Breakthrough ACTION project to combat COVID-19 and continue to shape them as the pandemic evolves. Specifically, messaging from the government and partners has sought to augment and sustain risk perception so that people understand that the disease is still present and a risk, even as the pattern of caseloads may seem to trend downwards. Messaging has also focused on capitalizing on the social influence of community and national leaders to highlight the importance of key prevention behaviors and normalize continuing certain behaviors such as mask wearing and social distancing. Even as the COVID-19 vaccine campaign began in Côte d’Ivoire, concurrent communication campaigns have emphasized the importance of sustaining other prevention behaviors, particularly considering the threat of new variants.

Based on these insights, the authors offer the following recommendations for actors involved in the challenge of improving adherence to COVID-19 prevention behaviors in an evolving and enduring pandemic:

- **Make behaviors that are perceived to be “antisocial” as easy as possible to practice.** Practices such as mask wearing, distancing, handwashing, or quarantine were acceptable during the pandemic’s early phase of heightened fear, but have diminished greatly in social acceptability over time, making it harder for even those conscious of the risks posed by COVID-19 to comply. To counter this, it is important that policymakers and those developing guidance try to make these preventative behaviors easier for people to do. For example, it may be important to consider how to reduce the financial barriers to purchasing masks and consider how to simplify the quarantine process for those potentially exposed.
- **Track and address rumors and conspiracy theories influencing risk perception.** No amount of promotion of prevention behaviors will be effective when the population believes that COVID-19 does not exist or that it does not affect certain groups. Misinformation continues to circulate on social media and in communities, and it is important to have a process for tracking and addressing them as they arise, using credible messengers and the channels most trusted by communities.
- **Address stigma attached to certain prevention behaviors.** Many Ivorians now associate mask wearing or post-exposure quarantine with COVID-19 infection. Messages should aim to reframe prevention behaviors as broadly protective against myriad diseases rather than as a sign of having or fearing COVID-19. Associating influential figures with social and behavior change messaging may help break stigma. Much as was done during the 2014 Ebola outbreak, when familiar forms of greeting such as handshaking and embracing had to be set aside, it is important that people see more socially difficult prevention behaviors as a show of caring for others.
- **Continue to highlight survivor stories to make the continued risk of COVID-19 infection real.** The challenge of declining risk perception is only likely to increase the longer the pandemic lingers. To achieve a meaningful protective effect through prevention behaviors people need to see the continued impact of infection with COVID-19 and the risks that it presents, especially for that portion of the population that may know someone who has had the disease.

Ultimately, reinforcing a narrative of cohesion and shared commitment is critical to building a social movement to end the pandemic. High-level leaders could do much to strengthen this framing but must be perceived as having integrity and “practicing what they preach” to be effective. Empathetic messaging that reinforces people’s collective power to protect one another and defeat the pandemic together appeals to the high value placed on social cohesion.

## Supporting information

S1 Questionnaire Inclusivity in global research. (DOCX)
