# Supplementary material for: “In our culture, if you quarantine someone, you stigmatize them”: Qualitative insights on barriers to observing COVID-19 prevention behaviors in Côte d’Ivoire
Source: PLOS Glob Public Health. 2022 Aug 24;2(8):e0000489. doi: 10.1371/journal.pgph.0000489 (PMC10022382; doi:10.1371/journal.pgph.0000489)
Supplement: S1 Questionnaire — (DOCX) [file pgph.0000489.s001.docx]

Inclusivity in global research

PLOS’ policy on inclusivity in global research aims to improve transparency in the reporting of research performed outside of researchers’ own country or community and ensures that PLOS publications reporting global research adhere to high standards for research ethics and authorship. Authors of relevant research articles may be asked to complete the questionnaire below, which outlines ethical, cultural, and scientific considerations specific to inclusivity in global research. This questionnaire may be requested when researchers have travelled to a different country to conduct research, if research uses samples collected in another country, research with Indigenous populations or their lands, or if research is on cultural artefacts. Researchers travelling to another country solely to use laboratory equipment will not normally be required to complete the questionnaire. However, the questionnaire can be requested at the journal’s discretion for any submission – if you have been requested to complete this questionnaire by the PLOS journal you submitted to, please do so.

Please complete the questionnaire below and include this as a Supporting Information file with your manuscript. Note that if your paper is accepted for publication, this checklist will be published with your article in the supporting information files. Please ensure that you reference the checklist in the main body of your manuscript. We suggest adding a subsection ‘Inclusivity in global research’ to your Methods section and adding the following sentence: “Additional information regarding the ethical, cultural, and scientific considerations specific to inclusivity in global research is included in the Supporting Information (SX Checklist)”

The questions have been designed to be applicable to a wide range of study types, and there are subsections for both human subjects research and non-human subjects research. If any of the questions are not relevant to your research please mark them as “N/A” as appropriate.

**Ethical considerations, permits and authorship**

*This section is applicable to all research types.*

Provide details as to who granted permissions and/or consent for the study to take place in the Methods section of your manuscript. This should include the names of **all** ethics boards, governmental organizations, community leaders or other bodies that provided approval for the study. If individuals provided approval refer to these people by their role or title but do not list their name(s).

The Ivoirian national research ethics committee (Comité National d’Éthique des Sciences de la Vie et de la Santé, CNESVS) approved the study, as did the Johns Hopkins Bloomberg School of Public Health Institutional Review Board [IRB#13757].

Reported on page number: 5

If there were any deviations from the study protocol after approval was obtained please provide details of these changes in the Methods section of your manuscript.
Did this study involve local collaborators that are residents of the country where the research was conducted or members of the community studied? If you do not have any authors from said communities, please provide an explanation for this below.

The study involved local collaborators and co-authors who are residents of Côte d’Ivoire.

Reported on page number: N/A

Everyone listed as an author should meet PLOS’ criteria for authorship and all individuals who meet these criteria should be included in the author byline, rather than the acknowledgements. Authorship criteria is based on the International Committee of Medical Journal Editors (ICMJE) Uniform Requirements for Manuscripts Submitted to Biomedical Journals - for further information please see here: <https://journals.plos.org/plosone/s/authorship>.

**Human subjects research (e.g. health research, medical research, cross-cultural psychology)**

Did you obtain written informed consent from a representative of the local community or region before the research took place? How did you establish who speaks for the community? Details of written informed consent obtained from study participants should be reported separately in the Methods section of your manuscript.

As the research took place in Abidjan (the capital and large urban center), we were confident that in working with the national program for orphans and vulnerable children (NPOVC) as well as local non-governmental organizations (NGOs), we would be able to connect well with communities. Recruitment of participants was done by three categories of focal points: the NPOVC, NGOs and a group of community health workers. All focal points were oriented by the CCP research team during a workshop. The research team oriented focal points on the study, its objectives, their roles, the risks associated with participation in the study, the benefits and the protection of personal information. They also emphasized the voluntary nature of participation in the study for anyone recruited. After this orientation, focal points moved to a time of recruitment. When potential participants expressed interest in participating and a verbal willingness to be contacted, he or she was put in contact with the research team, who went through the informed consent process individually, face-to-face. As these were adult participants who were not considered vulnerable, we did not ask for permission from community or household leaders for individual consent. Participants who were not interested in participating were not contacted by the research team.

How did members of the local community provide input on the aims of the research investigation, its methodology, and its anticipated outcome(s)?

During the elaboration of the support and collection tools, several meetings were held with the different stakeholders (NPOVC, the national technical working group on risk communication, and NGOs fighting against COVID) to discuss the study, its objectives and expected results. These meetings made it possible to take into account additional sub-populations that were not initially defined, to refine the methodology for recruiting participants and to take into account feedback in reporting the results. The meetings also allowed us to refine the methodology for approaching and recruiting participants for the study.

When engaging with the local community, how did you ensure that the informed consent documents and other materials could be understood by local stakeholders?

We conducted a multi-day, in person training with the data collectors who would take participants through the informed consent process. We discussed the language in the consent document, role played and practiced to ensure that the language was simple and clear. We ensured that all elements such as the purpose of the study, the role of the participant, the benefits and risks of participation, the protection of information, the respect of confidentiality, and the voluntary nature of participation were included in the consent information note using simple and clear language that was well understood by participants. Also, we gave the participants the opportunity to read the form themselves for those who could read and ask questions if they had questions of understanding before giving their agreement to participate in the study. It is when the participant declared to have understood and had no other question that the consent form was signed.

Will the findings of the research be made available in an understandable format to stakeholders in the community where the study was conducted (e.g. via a presentation, summary report, copies of publications, etc.)? Please provide details of how this will be achieved.

The results of the study were presented in several ways: A workshop was held in Abidjan to present the results to all stakeholders, the various focal points for the study, and other participants. Also, the study report was shared with stakeholders in the field which presents the main results of the study.

**Non-human subjects research using specimens/ animals collected as part of the study, or those housed in archival collections. Examples include archaeology, paleontology, botany and zoology.**

Did the permission you obtained from a local authority to perform the study include an agreement on access to outputs and benefit sharing? This may include procedures to enable fair distribution of the benefits and resources arising from the research performed. Please include any details of Prior Informed Consent and Benefit Sharing Agreements obtained. These may be required by field-specific regulations, for example the Convention on Biological Diversity (CBD) and the associated Nagoya Protocol.

N/A

If the material used in your study was imported, please A) provide the year it was imported and B) indicate whether permits were obtained to import/export the materials used, C) provide details of any permits obtained. If this information is not available, please indicate this.

N/A

If you used archival specimens, please state how the material used in your study was acquired by the institute it is held in and provide details of any permits obtained for the original excavations/ sample collection. If this information is not available, please indicate this.

N/A

How was the potential cultural significance of the materials collected in your study to local communities considered in your research design? Were Indigenous peoples and/or local researchers and institutions involved with archaeological excavations / collection of specimens? If so, please provide a description of their involvement.

N/A

If your manuscript includes photographs of human remains please indicate whether authors obtained permission from descendants or affiliated cultural communities to do so.

N/A
